# Galvanic vestibular stimulation increases novelty in free selection of manual actions

**DOI:** 10.3389/fnint.2013.00074

**Published:** 2013-11-05

**Authors:** Elisa R. Ferrè, Kobbina Arthur, Patrick Haggard

**Affiliations:** Institute of Cognitive Neuroscience, University College LondonLondon, UK

**Keywords:** galvanic vestibular stimulation, exploration, exploitation, novelty, hemispheric specialization, action selection

## Abstract

Making optimal choices in changing environments implies the ability to balance routine, exploitative patterns of behavior with novel, exploratory ones. We investigated whether galvanic vestibular stimulation (GVS) interferes with the balance between exploratory and exploitative behaviors in a free action selection task. Brief right-anodal and left-cathodal GVS or left-anodal and right-cathodal GVS were delivered at random to activate sensorimotor circuits in the left and right hemisphere, respectively. A sham stimulation condition was included. Participants endogenously generated sequences of possible actions, by freely choosing successive movements of the index or middle finger of the left or right hand. Left-anodal and right-cathodal GVS, which preferentially activates the vestibular projections in the right cerebral hemisphere, increased the novelty in action sequences, as measured by the number of runs in the sequences. In contrast, right-anodal and left-cathodal GVS decreased the number of runs. There was no evidence of GVS-induced spatial bias in action choices. Our results confirm previous reports showing a polarity-dependent effect of GVS on the balance between novel and routine responses, and thus between exploratory and exploitative behaviors.

## INTRODUCTION

The exploration and exploitation trade-off is a control dilemma that involves most adaptive behaviors, and is fundamental to the relation between organism and environment (Cohen et al., 2007). This dilemma is between choosing well-known options close to the expectations (*exploitation*) and choosing new options and possibly learning more (*exploration*) (Goschke, 2000). For example, in a restaurant you can *exploit* – choose your usual meal – or you can *explore* – try whatever dish you have not had before. Thus, exploitation involves perseveration and stereotyped behavior, while exploration involves discovering new possibilities and varying choices, and potentially larger rewards (Cohen et al., 2007).

Making optimal choices in an ever-changing world includes the ability to orient in the surrounding environment (Peacocke, 1983). Therefore, we suspected that vestibular inputs could contribute to the balance between exploration and exploitation. Vestibular information is crucial to determine the relation between the body and surrounding space, and therefore forms the starting point of almost all orienting behaviors. The semicircular canals detect rotational movements of the head, while the otolith organs detect gravitational and translational acceleration. These signals contribute to orienting by modulate somatosensory inputs (Vallar et al., 1990; Ferrè et al., 2011), controlling postural and balance stability (Day and Fitzpatrick, 2005), defining spatial parameters of movement (Karnath and Dieterich, 2006) and planning motor actions (Rode et al., 1998). Primate studies revealed that vestibular input does not project to a primary vestibular cortex, but to a network of multimodal areas, notably the parieto-insular vestibular cortex (PIVC; Guldin and Grüsser, 1998). The PIVC consists of the posterior insula/retroinsular cortex in the upper or lower banks of the lateral sulcus (Guldin and Grüsser, 1998). Recent functional neuroimaging studies in humans have shown that artificial vestibular stimulation, whether galvanic, caloric, or sound-induced, activates a wide range of multimodal areas, involving the parietal and insular cortices and also the temporal cortex, putamen, and thalamus (Lopez et al., 2012). This conjunction of anatomical projections and physiological activations are broadly consistent with the view that the vestibular system acts as a basic reference system for other sensorimotor representations.

We recently found that vestibular inputs contribute to the balance between exploration and exploitation in a random number generation task (Ferrè et al., 2013). This effect was hemisphere-specific. Left-anodal and right-cathodal galvanic vestibular stimulation (GVS), which primarily activates the right hemisphere, increased randomness of sequences compared to right-anodal and left-cathodal GVS. However, vestibular stimulation also produces spatial, attentional, and arousing effects. Therefore, to investigate whether vestibular stimulation truly involves modulation of behavior selection, rather than these other independent but frequently-associated functions, we have investigated whether GVS interferes with the generation of novel vs. routinized behaviors in an endogenous action selection task. In this task, participants endogenously generated a sequence of movements, by freely choosing between four possible actions, involving the index or middle finger of the left or right hand.

In discussing endogenous action selection, it is important to distinguish between “*free selection*” of single action, and generation of “*action patterns*” which exist only in the context of a sequence of actions. The trade-off between exploration and exploitation refers to sequences, or *runs, *of behavior in which the endogenous choice of any individual action is determined partly by what the participant has done before. Extreme exploitation might involve constantly repeating one action or action sequence, while extreme exploration might involve complete randomness. A pattern of action selection can therefore be analyzed quantitatively, and placed on a continuum between stereotypy and novelty. Based on our previous findings with random number generation (Ferrè et al., 2013), we hypothesized that GVS might have a polarity-dependent effect on action selection, with left-anodal and right-cathodal GVS promoting randomness/exploration rather than stereotypy/exploitation (Ferrè et al., 2013). However, it was unclear whether similar organization of vestibular influences on behavior would occur for bimanual movements as for generation of symbolic items such as numbers.

We delivered binaural GVS between the mastoids, to activate peripheral vestibular organs, i.e., both otoliths and semicircular canal afferents (Stephan et al., 2005). This induces a polarity-dependent “*virtual rotation vector*” (Day and Fitzpatrick, 2005) which can influence orientation perception and posture. More surprisingly, GVS also influences sensory and cognitive functions (Utz et al., 2010). The effects are polarity dependent. Left-anodal and right-cathodal GVS mimics an inhibition of the left and an activation of the right ear vestibular peripheral organs, decreasing the firing rate of the vestibular nerve on the left side and increasing it on the right side (Goldberg et al., 1984; Fitzpatrick and Day, 2004). In contrast, right-anodal and left-cathodal GVS induces the opposite effect. Neuroimaging studies have revealed asymmetrical cortical vestibular projections, suggesting that the core region of the vestibular network is primarily located in the non-dominant hemisphere in right-handed subjects (Dieterich et al., 2003).

Clinical observations have reported strong effects induced by vestibular stimulation on spatial attention in brain-damaged patients (Rubens, 1985; Utz et al., 2011). Recently, a contribution of vestibular information to the allocation of attention has also been****suggested in healthy volunteers (Figliozzi et al., 2005). We therefore hypothesized that GVS could have spatial effects on the generation of free actions. In particular, given the specialization of the right hemisphere for spatial responding, GVS-induced activation of vestibular projections in the right hemisphere might cause an attentional shift toward the left side of the space or body (Rubens, 1985), and thus a preference for the left hand, or the leftmost digit, in action selection.

## MATERIALS AND METHODS

### PARTICIPANTS

Sixteen naïve right-handed paid participants volunteered (10 male, mean age 24.7 years ± 5.08 SD). Subjects with a history of visual, vestibular, or auditory disorders were excluded. Informed consent was obtained prior to participation in the experiment. The experimental protocol was approved by University College London research ethics committee. The study was designed according to ethical standards of the Declaration of Helsinki.

### GALVANIC VESTIBULAR STIMULATION

**FIGURE 1:**
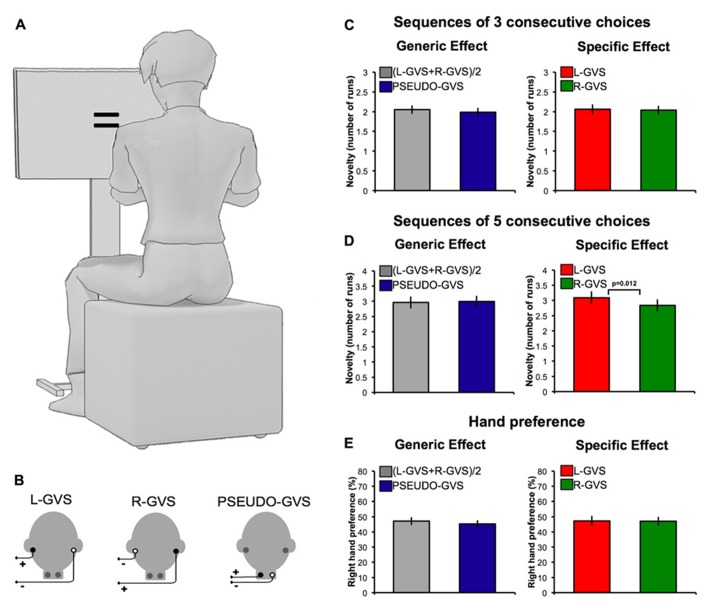
**Effects of GVS on spatial bias and novelty. (A)** Experimental set-up. **(B)** GVS polarities and electrodes configurations. **(C)** Number of runs in sequences of three successive free-choices in each experimental condition. **(D)** Number of runs in sequences of five successive free-choices in each experimental condition. **(E)** Preference for right hand as a function of GVS condition.

Bipolar GVS was used to deliver a boxcar pulse of 1 mA via a commercial stimulator (Good Vibrations Engineering Ltd., Nobleton, ON, Canada). Carbon rubber electrodes (area 10 cm^2^) were placed binaurally over the mastoid processes and fixed in place with adhesive tape. The areas of application were first cleaned with cotton wool soaked in surgical spirit, and electrode gel was applied to reduce the impedance. Left-anodal and right-cathodal (“L-GVS”) was used to predominantly stimulates the right hemisphere, while the inverse polarity, left-cathodal and right-anodal configuration, “R-GVS,” was used to predominantly stimulate the left hemisphere (**Figure 1B**). A “PSEUDO-GVS” sham stimulation, based on that used by Lopez et al. (2010), was applied using left-anodal and right-cathodal stimulation of the neck, 5 cm below the mastoids (**Figure 1B**). This causes a similar tingling skin sensation to real GVS, and therefore functions as a control for non-specific alerting effects.

### STIMULI AND PROCEDURE

Data from each participant was gathered in a single session. Verbal and written instructions about the task were given to participants at the beginning of the session. Participants sat 50 cm from a screen and made sequences of fingers movements initiated by auditory and visual cues during GVS or PSEUDO-GVS stimulation. Electrodes for GVS and PSEUDO-GVS were placed at the beginning of the session and remained in place for the entire duration of the experiment. The electrodes and the polarity of stimulation were selected under randomized, double-blind, computer control at the start of each block.

A total of 15 blocks were administered, five for each type of stimulation (L-GVS, R-GVS, and PSEUDO-GVS). The order was randomized across participants. Before the beginning of the task, participants received a number of short training blocks. These blocks introduced and familiarized participants with the visual and auditory cues. No vestibular or sham stimulation was delivered during the training.

Each block comprised 21 trials in random order. Each trial began with a symbol “L,” “R,” or “=,” on the center of the screen. The symbol instructed participants which hand to respond with (“=” meaning that the participant could press a button with a hand of her choice). The auditory signals referred to the *finger* for responding. A high frequency beep instructed participants to use the index finger, a low frequency beep signaled the middle finger, and a mid frequency beep signaled a free choice. The visual stimulus appeared before the auditory cue (**Table 1** and **Figure 1A**). The participant was asked to monitor the visual and auditory cues and make an appropriate keypress. For example, if the auditory tone for the index finger was heard and the “=” stimulus displayed, the participant should press a button with the index finger of whichever hand she chose. If the “free” tone was heard and a “L” was displayed on the screen, she should move either the middle or index finger of her left hand. If the “free” tone was heard and the “=” was displayed, the choice of both finger and hand was entirely free. Thus each trial was composed of a visual cue and the corresponding sequence of auditory cues. Three different trial lengths were presented, at random: single choice trial, sequences of three consecutive choices and sequences of five consecutive choices (**Table 1**).

**Table 1:** Experimental conditions in the free action generation task.

	Visual cue = hand	Auditory cue = finger
Single choice	Left	Index	Middle	Free
	Right	Index	Middle	Free
	**Free**	Index	Middle	**Free**
Sequences of 3	Left	Index	Middle	Free [-1pc]
consecutive choices	Right	Index	Middle	Free
	**Free**	Index	Middle	**Free**
Sequences of 5	Left	Index	Middle	Free [-1pc]
consecutive choices	Right	Index	Middle	Free
	**Free**	Index	Middle	**Free**

*Possible combinations of movements during the free action generation task.*

Galvanic vestibular stimulation or PESUDO-GVS began 2,000 ms before each sequence, and ended 200 ms after the last beep. The inter-tone interval ranged between 1,000 and 1,400 ms randomly and uniformly. This cadence was adopted to discourage purely rhythmic responses and to maintain response times. The average inter-tone interval was decided after a pilot study, suggesting that 1,200 ms was sufficiently long to allow quick decisions after each imperative stimulus, whilst preventing pre-decision of which response to make.

Responses were collected via a custom keypad. The keypad was held vertically facing away from the participant (**Figure 1A**) to exclude the possibility that the lateral spatial position of the response key could influence finger choice. The index and middle fingers of left and right hands remained on the keypad throughout. Participants were instructed to maintain contact, and depress an appropriate key, within 800 ms of the visual and auditory stimuli. Ill-timed or multiple responses were also recorded. Participants were instructed to respond as quickly and accurately as possible. Participants were also encouraged to respond spontaneously and without pre-decision when a free choice of finger and/or hand was indicated.

### DATA ANALYSIS

Because our interest focussed on vestibular modulation of action selection, we analyzed only responses obtained in the free choice conditions. Based on the distinction made above, we had different hypotheses about selection of a single action, versus sequential patterns of several actions. In particular, the trade-off between exploration and exploitation reflects the relation between each action in a sequence and the previous actions. This determines whether the sequence reflects a routinized or innovative action patterns. This relation is absent in a single instance of free action selection, and becomes progressively more important as the run of successive free actions lengthens. Therefore, we analyzed the sequences of three and five “free” consecutive choices, and we predicted that any effects of vestibular input on novelty of action choices would be stronger for longer sequences. Novelty was defined by the number of adjacent identical elements. For example, the sequence “AAAAA” comprises one run, while the “AAAAB” comprises two runs. Thus, the maximum and minimum number of runs, given five successive choices between four possible actions, are five and one, respectively.

Trials involving just one “free” choice were analyzed separately, to identify whether vestibular input generated a preference for one particular response. The percentage of right hand choices in response to free stimuli (“=” visual cues and the “free” tone) was calculated. We hypothesized that right-anodal and left-cathodal stimulation (R-GVS) would induce a right hand preference compared to left-anodal and right-cathodal stimulation (L-GVS). That is, this analysis focussed on spatial biases in individual action selection, rather than on sequential action patterns.

The number of runs in three and five-choices trials and percentage of right hand preferences were estimated for each sequence, and averaged for the three experimental conditions: L-GVS, R-GVS, and PSEUDO-GVS. We hypothesized that vestibular stimulation might influence our variables in two distinct ways (Ferrè et al., 2013), and we tested these hypotheses as planned contrasts. First, *any* activation of the vestibular system might influence free action selection independent of polarity and hemispheric effects. To test this *generic hypothesis*, we compared the average of the L-GVS and R-GVS conditions to the PSEUDO-GVS condition, for each dependent variable. Second, we hypothesized that the effects of vestibular stimulation could be *specific* to the hemisphere activated, and would therefore differ between L-GVS and R-GVS conditions.

## RESULTS

The mean data in each condition are shown in **Table 2**.

**Table 2:** Mean scores in each stimulation condition.

		Stimulation condition		
		L-GVS	R-GVS	PSEUDO-GVS
Measures of spatial bias	Right hand preference (%)	47.25	47.06	45.28
		(13.38)	(11.61)	(9.80)
Measures of novelty	Number of runs:	2.06	2.04	1.99
	Sequences of 3 consecutive choices	(0.49)	(0.44)	(0.44)
	Number of runs:	3.09	2.83	2.99
	Sequences of 5 consecutive choices	(0.85)	(0.78)	(0.77)

*Measures of spatial bias and novelty (mean scores, SD) in L-GVS, R-GVS, and PSEUDO-GVS.*

### GENERIC VESTIBULAR EFFECTS ON NOVEL ACTION SELECTION

The generic vestibular effect, defined as (L-GVS + R-GVS)/2, was compared to the PSEUDO-GVS condition. A 2 × 2 ANOVA with stimulation ((L-GVS + R-GVS)/2 vs. PSEUDO-GVS) and sequence length (sequences of three consecutive choices vs. sequences of five consecutive choices) was performed. This analysis revealed a predictable main effect of sequence length (*F*_(1,15)_ = 92.171, *p* < 0.001), indicating more runs of a single action choice in sequences of five consecutive choices compared to sequences of three consecutive choices, as one might expect (**Figures 1C,D**). No significant main effect of stimulation (*F*_(1,15)_ = 0.086, *p* = 0.773) or interactions between the factors (*F*_(1,15)_ = 1.337, *p* = 0.266) was found (**Figures 1C,D**).

### HEMISPHERE-SPECIFIC VESTIBULAR EFFECTS ON NOVELTY GENERATION

We next compared L-GVS and R-GVS conditions. A 2 × 2 ANOVA with stimulation (L-GVS vs. R-GVS) and sequence length (sequences of three consecutive choices vs. sequences of five consecutive choices) was performed. This analysis revealed a main effect of sequence length (*F*_(1,15)_ = 62.672, *p* < 0.001). The effect of stimulation showed a trend toward conventional statistical significance**(*F*_(1,15)_ = 3.142, p = *0.097*)*.* A significant interaction between stimulation and sequence length has been found (*F*_(1,15)_ = 9.800, *p* = 0.007). To further investigate this interaction, we directly compared L-GVS and R-GVS sequences of three and five consecutive choices. No significant difference was found in sequences of three consecutive choices (*t*_(15)_ = 0.251, *p* = 0.806; **Figure 1C**), but a significant effect emerged for sequences of five choices (*t*_(15)_ = 2.86, *p* = 0.012; **Figure 1D**). To investigate whether this effect reflected a benefit of L-GVS or a cost of R-GVS, we additionally compared each individual stimulation condition to PSEUDO-GVS. Neither GVS condition was significantly different from the PSEUDO-GVS (*p* > 0.05), suggesting that the effect lay in an approximately equal balance between the two hemispheric stimulations.

### GENERIC AND SPECIFIC EFFECTS ON HAND PREFERENCE

Investigation of spatial effects of GVS, measured as preference for right hand actions on free-choice trials, did not reveal generic (*t*_(15)_ = 0.756, *p* = 0.461) or specific vestibular effects (*t*_(15)_ = 0.054, *p* = 0.958; **Figure 1E**).

### STIMULATION EFFECTS ON TASK ERRORS

To investigate whether GVS has a specific effect on motor responses, we analyzed errors made by participants during the endogenous actions generation task. Ill-timed and multiple responses were counted across the three experimental conditions (L-GVS, R-GVS, PSEUDO-GVS). Since this analysis aimed to investigate differences between L-GVS, R-GVS, and PSEUDO-GVS on error rate, all free choice trials (one choice, sequences of three consecutive choices and of five consecutive choices) were considered. The percentage of errors was: L-GVS 4.03%; R-GVS 4.23%, PSEUDO-GVS 6.78%).

A 3 × 3 ANOVA on error rate with stimulation (L-GVS, R-GVS, PSEUDO-GVS) and sequence length (one choice, three consecutive choices and five consecutive choices) was performed. This analysis revealed a main effect of sequence length (*F*_(2,30)_ = 6.396, *p* = 0.005). Participants showed the tendency to commit more errors during single choices trials compared to trials of three consecutive choices (*t*_(15)_ = 2.374, *p* = 0.031) and five consecutive choices (*t*_(15)_ = 3.354, *p* = 0.004). Importantly, no significant effect of stimulation (*F*_(2,30)_ = 1.785, *p* = 0.185) or interaction between stimulation and sequence length (*F*_(4,60)_ = 0.743, *p* = 0.567) was found.

## DISCUSSION

Here we demonstrated that vestibular input in *general* did not influence the generation of novelty in a free selection of manual responses. In contrast, *specific* polarities of vestibular input, associated with hemisphere-specific activations, had significantly different effects on free selection. Left-anodal and right-cathodal GVS increased novelty of action selection, relative to right-anodal and left-cathodal GVS, which decreased novelty. In other words, left-anodal and right-cathodal boosted the selection of different actions, shifting more often between fingers and hands movements, while right-cathodal and left-anodal promoted the repetition of the same finger movement.

GVS polarity-dependent differences in postural, sensorimotor, and cognitive functions have been demonstrated both in healthy volunteers and in brain damaged patients (Utz et al., 2010). Fink et al. (2003) used fMRI to study the effects of bipolar GVS. They found that left-anodal and right-cathodal GVS produced unilateral activation of the right hemisphere vestibular projections, while the opposite polarity, i.e., left-cathodal and right-anodal GVS, activated both left and right hemispheres (Fink et al., 2003). These results are coherent with the asymmetrical cortical vestibular representation in the right hemisphere in right-handed subjects (Bense et al., 2001; Suzuki et al., 2001; Dieterich et al., 2003; Janzen et al., 2008). Importantly, the observed hemispheric-specific effects might arise because of this cortical asymmetry, or because one polarity of GVS has stronger effects in the brain.

We suggest that the difference between L-GVS and R-GVS in action selection reflects the activation by vestibular input of a large-scale hemispheric network for behavioral control. Left and right cerebral hemispheres play different roles in novelty and cognitive routine. Goldberg and Costa (1981) formulated a *novelty-routinization* model, suggesting a strong hemispheric specialization in behavioral control. The right cerebral hemisphere is responsible for exploratory processing of new cognitive situations, particularly in the absence of any pre-existing cognitive strategy (Goldberg and Costa, 1981; Goldberg et al., 1994; Goldberg and Podell, 1995). In contrast, the left hemisphere is specialized for processing of pre-existing representation and routine cognitive strategies. Thus, right brain-damaged patients showed more repetitive behavior than patients with comparable lesions in the left hemisphere (Sandson and Albert, 1987; Goldberg et al., 1994). We suggest that the hemispheric-specific activations induced by GVS similarly influence the balance between generative and repetitive behaviors. Left-anodal and right-cathodal (L-GVS) would boost the selection of movements based on exploration, by activating the right hemisphere, while R-GVS would reduce novelty by promoting stereotyped behaviors controlled by the left hemisphere. Given the multimodal nature of vestibular cortical projections, we cannot exclude the possibility that vestibular signals reach specific frontal or parietal areas involved in the generation of motor planning. Thus, it remains unclear if our results reflect activations which produce a diffuse imbalance *between* hemispheres, or whether specific activations *within* each hemisphere are responsible.

Our results confirmed recent findings of GVS effects on random number generation (Ferrè et al., 2013). To that extent, they suggest a general, task-independent vestibular contribution to novel behavior. Left-anodal and right-cathodal GVS increased randomness compared to right-anodal and left-cathodal GVS, which decreased it (Ferrè et al., 2013). The polarity-specific effects of GVS were therefore consistent for the random number generation studied previously, and the free selection of manual responses studied here. This is particularly significant, given that the cortical areas involved in number generation and action selection are at least partly different. The generation of random numbers activated the dorsolateral prefrontal cortex, the lateral premotor cortex, the anterior cingulated, and the inferior and superior parietal cortex (Jahanshahi et al., 1998, 2000; Daniels et al., 2003). In contrast, endogenous action selection involves more medial areas, as the premotor cortex, the supplementary motor area, the intraparietal sulcus, the cingulated gyrus (Grezes and Decety, 2001). Given this similarity of GVS effects across output modalities, we suggest that vestibular stimulation may influence high-level features of action control, that go beyond a specific output system or a single cortical programming center. The balance between innovation/exploration and perseveration/exploitation might be one such high-level parameter.

Fink et al. (2009a,b) studied changes in brain activity during novel, or creative, processing such as the generation of alternative behaviors. Individuals who scored higher on indices of originality had a stronger EEG alpha rhythm in the right than in the left hemisphere, while less original individuals showed no hemispheric asymmetry (Fink et al., 2009a). These results have been replicated using a range of different testing paradigms (Fink et al., 2009a,b) suggesting a modality independent effect of novelty on neural processing. Thus, the content of representations and behavior generated may not be crucial: almost any behavior or choice can be performed in a novel way, or repeated in a routine, preservative or stereotyped way. Our data also support this hypothesis and provide additional evidence that left and right cerebral hemispheres are differentially involved in novelty and cognitive routine generation.

Further, effects of GVS interacted with sequence length. This is also consistent with an account based on generation of novel behavior. Sequence length influences randomness for simple reasons of sampling, since the probability of generating novel choices inevitably increases with the length of the sequence (Schulz et al., 2012). Nevertheless, sequence length also affects randomness judgements, but in the opposite direction. Participants judge short sequences as more random than long sequences. Tversky and Kahneman (1971, 1974) suggested that participants try to produce a sequence of choices that is representative of a random process over a short section of behaviors. Thus, the desire to produce this representative sequence influences short sequences rather than long sequences. In line with this notion, short sequences are barely influenced by other factors. This would account for the interaction with sequence length in our data.

Although several clinical observations reported strong effects induced by vestibular stimulation in visuo-spatial attention (Utz et al., 2011), our data did not show any evidence of spatial bias. Galvanic vestibular stimulation was previously shown to interfere with spatial processing in healthy participants during spatial tasks (Dilda et al., 2012). Similarly, Figliozzi et al. (2005) demonstrated that vestibular inputs could produce spatiotopic shifts of attention. We thus hypothesized that vestibular input might shift spatial attention toward one side of the body, as a result of activating the contralateral hemisphere. In particular, attentional accounts would predict that left-anodal and right-cathodal should cause a preference for selecting the left hand. However, our data did not support this prediction. Further studies are needed to clarify the role of vestibular inputs in higher order spatial and attentional processing, in particular related to body representation. In the meantime, two possibilities exist. First, GVS may be too weak to cause spatial effects in healthy participants. Second, previous effects of vestibular stimulation in patients (Rubens, 1985) may have overestimated the vestibular role in spatial attention.

In conclusion, our results confirm previous reports showing polarity-dependent effects of GVS on the balance between novel and routine responses, and thus between exploratory and exploitative behaviors. We suggest that the vestibular-mediated balancing between exploitation and exploration may be a crucial, but neglected, element of the brain’s capacity to interact with the environment.

## Conflict of Interest Statement

The authors declare that the research was conducted in the absence of any commercial or financial relationships that could be construed as a potential conflict of interest.

## Abbreviations

GVS: galvanic vestibular stimulation
PIVC: parieto insular vestibular cortex
